# A hypoxic ticket to the bone metastatic niche

**DOI:** 10.1186/s13058-015-0635-7

**Published:** 2015-09-04

**Authors:** Sakari Vanharanta

**Affiliations:** MRC Cancer Unit, University of Cambridge, Hutchison/MRC Research Centre, Cambridge, CB2 0XZ UK

## Abstract

Hypoxia is a well-characterized driver of aggressive cancer phenotypes, including metastasis. Accumulating evidence suggests that, in addition to having local effects, the consequences of tumour hypoxia can be systemic, leading to the formation of pre-metastatic niches that can later foster metastatic colonization in distant organs. Recent findings have demonstrated that such niches can also form in the bone, possibly revealing new avenues for therapeutic intervention.

Cancer metastasis is a complex process that consists of a series of biological phenomena that eventually leads to the formation of secondary tumours in distant organs. Even though often pictured as an orderly cascade of events akin to organismal development, at the cellular level metastatic tumours emerge from a disorderly process whereby the dissemination of millions of cells can eventually lead to the development of only few secondary tumours [1]. Yet different tumour types have tendencies to colonize specific organs, suggesting that underneath the chaos there are principles of order. One such principle is that metastases need a fertile soil, that is, a hospitable microenvironment that supports cancer cells at the early steps of metastatic colonization [2].

The molecular definition of a fertile metastatic soil remains in most cases elusive. It could consist of stem cell or other niches that provide cancer cells with the required survival signals in a tissue-specific manner [3]. However, it is clear that beyond pre-existing microenvironments, cancer cells also need to alter their molecular surroundings in order to metastasize [4]. This can be achieved through local cancer cell effects on their immediate microenvironment, but also via systemic effects that modulate the molecular milieu in distant organs, resulting in the formation of pre-metastatic niches in which the efficiency of metastatic colonization is enhanced. Pre-metastatic niches have been described in various experimental models, mostly in the lung and other soft tissues, and they are often characterized by the involvement of various immune cell types [5]. How and when to therapeutically target the many forms of cancer cell-niche interactions at different steps of metastatic cancer progression remain critical open questions.

In a recent study, Cox and colleagues [6] add a new chapter to our understanding of metastatic niches by demonstrating that oestrogen receptor-negative (ER–) breast cancer cells with increased potential to form bone metastases secrete the enzyme lysyl oxidase (LOX) in response to hypoxia, leading to formation of osteolytic bone alterations already before cancer cells reach the bone. Administering cancer cell-conditioned media was sufficient for the induction of osteolysis, and this enhanced bone colonization in a LOX-dependent manner. Finally, as bisphosphonate treatment is a well-established strategy to fight osteoporosis, that is, reduced bone density, the authors show that bisphosphonates can also reverse the formation of osteolytic pre-metastatic niches and bone metastases in their model systems.

The pathobiology of osteolytic bone metastases has been widely studied, revealing a central role for the RANKL-RANK system that controls osteoclast maturation [7]. Specifically, various tumour cell-derived factors, such as interleukin 6 and PTHrP (parathyroid hormone-related protein), induce RANKL expression in osteoblasts, resulting in RANK-mediated activation of osteoclasts and consequent bone resorption. This in turn releases growth and other factors from the bone matrix, stimulating the cancer cells to produce more factors that further stimulate osteoclasts. Interestingly, the results by Cox and colleagues now suggest that this osteolytic vicious cycle may be kick-started already before cancer cells arrive and that this can be independent of RANKL. The authors show that recombinant LOX is able to activate the transcription factor NFATC1 (nuclear factor of activated T-cells, cytoplasmic 1), a key driver of osteoclast maturation, but the molecular pathway through which the extracellular enzyme LOX transmits a nuclear signal was not dissected in detail.

Based on initial bioinformatic analysis of clinical data sets, Cox and colleagues focus on ER– breast cancer in their experimental work. The data suggest, however, that many ER+ breast cancers also express high levels of LOX, even though this is not associated with bone metastases. Given the tendency of ER+ breast cancers to metastasize to the bone [8], it would be interesting and clinically relevant to know whether ER+ and ER– breast cancer cells use different molecular mechanisms to induce osteolysis.

A conclusion highlighted by Cox and colleagues is that LOX expression in ER– breast cancer could be a useful biomarker for increased bone metastasis risk as well as for the identification of patients for bisphosphonate treatment in the adjuvant setting. The use of bisphosphonates in breast cancer patients has a long history, but the results have been inconclusive [9, 10]. Moreover, it is not clear how much of the potential benefit comes from actual antitumour effects and how much is related to a reduction in fractures and consequent mortality [9]. From a biological perspective, the results by Cox and colleagues are exciting and they give novel insight into the molecular mechanisms of bone metastasis. From a clinical perspective, however, it is not clear how targeting the pre-metastatic niche could be achieved in practice. Cancer cells have often already disseminated at the time of diagnosis. Targeting niche interactions that support developing bone metastases could therefore be a more efficient way forward.

## Abbreviations

ER: Oestrogen receptor
LOX: Lysyl oxidase
RANK: Receptor activator of nuclear factor κB
RANKL: Receptor activator of nuclear factor κB ligand

## References

[1] Vanharanta S, Massague J (2013). Origins of metastatic traits. Cancer Cell.

[2] Fidler IJ (2003). The pathogenesis of cancer metastasis: the ‘seed and soil’ hypothesis revisited. Nat Rev Cancer.

[3] Lander AD, Kimble J, Clevers H, Fuchs E, Montarras D, Buckingham M (2012). What does the concept of the stem cell niche really mean today?. BMC Biol.

[4] Oskarsson T, Batlle E, Massague J (2014). Metastatic stem cells: sources, niches, and vital pathways. Cell Stem Cell.

[5] Sceneay J, Smyth MJ, Moller A (2013). The pre-metastatic niche: finding common ground. Cancer Metastasis Rev.

[6] Cox TR, Rumney RM, Schoof EM, Perryman L, Hoye AM, Agrawal A (2015). The hypoxic cancer secretome induces pre-metastatic bone lesions through lysyl oxidase. Nature.

[7] Mundy GR (2002). Metastasis to bone: causes, consequences and therapeutic opportunities. Nat Rev Cancer.

[8] Kennecke H, Yerushalmi R, Woods R, Cheang MC, Voduc D, Speers CH (2010). Metastatic behavior of breast cancer subtypes. J Clin Oncol.

[9] Valachis A, Polyzos NP, Coleman RE, Gnant M, Eidtmann H, Brufsky AM (2013). Adjuvant therapy with zoledronic acid in patients with breast cancer: a systematic review and meta-analysis. Oncologist.

[10] Yan T, Yin W, Zhou Q, Zhou L, Jiang Y, Du Y (2012). The efficacy of zoledronic acid in breast cancer adjuvant therapy: a meta-analysis of randomised controlled trials. Eur J Cancer.

